# Rubber-like PTFE Thin Coatings Deposited by Pulsed Electron Beam Deposition (PED) Method

**DOI:** 10.3390/polym16091205

**Published:** 2024-04-25

**Authors:** Agata Niemczyk, Roman Jędrzejewski, Joanna Piwowarczyk, Jolanta Baranowska

**Affiliations:** 1 Department of Materials Technology, Faculty of Mechanical Engineering and Mechatronics, West Pomeranian University of Technology in Szczecin, Piastów Avenue 19, 70-310 Szczecin, Poland; 2 Łukasiewicz Research Network—PORT Polish Center for Technology Development, Stabłowicka 147, 54-066 Wrocław, Poland

**Keywords:** pulsed electron deposition, coatings, crosslinked PTFE, amorphous, infrared spectroscopy, scratch test

## Abstract

PTFE coatings were manufactured using the pulsed electron beam deposition (PED) technique and deposited on Si substrates. The deposition was carried out at constant parameters: temperature 24 °C, discharge voltages 12 kV, and 5000 electron pulses with a pulse frequency of 5 Hz. Nitrogen was used as the background gas. The gas pressure varied from 3 to 11 mTorr. The coating adhesion was evaluated using micro scratch testing and the residual scratch morphology was characterized by atomic force microscopy. Detailed studies of the chemical and physical structure were conducted using infrared spectroscopy and X-ray diffraction. These analyses were then correlated with the mechanical response of the coatings observed during the scratch tests. Drawing upon a review of the literature concerning energetic beam interactions with PTFE material, hypotheses were posed to explain why only specific conditions of the PED process yielded PTFE coatings with rubber-like properties.

## 1. Introduction

In recent years, PTFE coatings have been extensively produced using various physical vapor deposition (PVD) techniques [1,2,3]. This trend arises from the advantage offered by these techniques, wherein the polymer material does not require to be dissolved during the coating production, which is an essential feature in the case of insoluble PTFE. Depending on the specific PVD technique used, the resulting PTFE films exhibit variations in thickness, roughness, chemical structure (i.e., C:F ratio), and degree of crystallinity [3,4,5]. However, regardless of the chosen PVD technique, a common characteristic is the high crystallinity of the PTFE coatings. Highly ordered and highly crystalline thin coatings are created by successive material deposition and attachment of CF_2_ groups, which, due to the size of the fluorine atom, forces a linear chain structure to form [6,7,8]. Such highly crystalline PTFE coatings find particular applications in optics and optoelectronics [9]. Nevertheless, although their crystallinity and other physical properties are often favourable, PTFE coatings suffer from drawbacks such as pronounced softness, susceptibility to scratches, and often limited adhesion to substrates.

Pulsed electron beam deposition (PED) represents a relatively recent physical vapor deposition technique in coating applications. This method facilitates the deposition of transparent or highly reflective materials at a cost-effective and efficient rate [10]. While PED has already demonstrated its significance in PVD techniques for inorganic material deposition, its potential within the domain of polymeric materials has not yet been fully recognized, with primary efforts currently directed toward PTFE coatings [11,12].

The accepted strategy for the deposition of polymeric materials using high-energy pulsed beam methods involves conducting the process under conditions near the ablation threshold of the material. This is primarily intended to prevent the degradation of the covalent bonds in the polymer material, which are inherently susceptible and prone to breakage [13,14,15]. In PED processes, adjusting the voltage applied to the electron gun (accelerating potential) directly influences the beam power density, thus setting this parameter is the most commonly employed method for establishing the conditions for PTFE coating deposition [15]. Extreme voltage values, whether low or high, results in a lower electron beam power density. Low voltages directly reduce the energy of the electron beam, while high voltages lead to significantly greater accelerating potential and target material penetration, thus also resulting in lower energy density [10,15]. Altering the voltage in the electron gun also changes the nature of the electron interaction with the ablated material, similar to how changing the laser wavelength affects generated photons and their interaction with the material.

A second method, in which the gas pressure in the chamber is adjusted to the accelerating potential is therefore appealing. Gas plays a crucial role in the PED method as it is essential for generating and propagating the electron beam with a high electron density. The gas undergoes ionization, counteracting strong space charge repulsion and preventing excessive expansion of the electron beam. For a given voltage, there is an optimum quantity of gas molecules (expressed in pressure) that enables complete compensation of the negative charge of electrons, resulting in the most focused beam and the highest beam fluence [15]. Reducing the pressure below this optimum value leads to an incomplete balance of the negative charge, causing beam fluctuations, broadening, and a reduction in fluence. When the gas pressure exceeds the optimal pressure range, the increased number of gas molecules intensifies scattering collisions with electrons, resulting in beam widening and a subsequent reduction in beam fluence [16]. Moreover, depending on the pressure used, the topography of the obtained coatings may also change, particularly the roughness and the presence of particles [16,17,18]. Therefore, when optimizing the PED process, it is worthwhile to consider not only different voltages but also different gas pressures.

Since one of the most crucial aspects of coatings, especially challenging with hydrophobic polymer coatings, is strong adhesion to the substrate, we conducted scratch tests on our coatings as a continuation of our research [19,20]. During our previous studies, we observed unconventional behaviour in the mechanical properties of the coatings, similar to that of typical elastomeric materials. This led us to hypothesize that the deposited PTFE material is in an amorphous and likely crosslinked form, which is generally difficult to achieve for PTFE. To date, amorphous and crosslinked PTFE has only been achieved through bulk material radiation at elevated temperatures, under specific conditions. Radiated PTFE is characterized by low chemical structure alterations and a notably distinct mechanical behaviour. In terms of coatings, only amorphous coatings have been obtained by depositing previously chemically modified amorphous PTFE—Teflon AF [21,22]. However, being amorphous but lacking chemical crosslinking, it likely did not exhibit elastomeric properties.

Our current research aimed to elucidate the unconventional mechanical behaviour of the coatings in relation to their structure, specifically focusing on crystallinity and chemical crosslinking. To achieve this, we conducted detailed studies of the chemical and physical structure using infrared spectroscopy and X-ray diffraction. These analyses were then correlated with the mechanical response of the coatings observed during scratch tests. Drawing upon a review of the literature concerning energetic beam interactions with PTFE material, we have formulated hypotheses to explain why only specific conditions of the PED process yield PTFE coatings with rubber-like properties.

## 2. Materials and Methods

PTFE coatings were deposited using the PED system (NEOCERA, Inc., Beltsville, MD, USA) following procedures detailed elsewhere [19,20]. The PTFE coatings were deposited on Si substrates at chamber pressures of 3, 5, and 11 mTorr, at room temperature. The electron source operated at 12 kV, with a repetition rate of 5 Hz and 5000 pulses (preceded by 2000 pre-ablation pulses). The distance between the target and substrate was set at 80 mm, and the distance between the ceramic end of electron gun and the PTFE target at 18 mm. A 50 mm diameter bulk PTFE disk with purity 99% (Tarflen^®^, P.H.U. SZCZEL-PLAST S.C., Mikołajów, Poland) was used as the target.

The PTFE structure was analysed by FT-IR spectroscopy (Lumos, Bruker, Billerica, MA, USA). Each spectrum consisted of 64 scans at a resolution of 4 cm^−1^ with an air background and was corrected for CO_2_ and H_2_O. Spectra were baseline-corrected and collected in the wave number range 3000–600 cm^−1^. To quantify changes in the individual band intensities to define all bonds in the material, deconvolution of the 1800–800 cm^−1^ region was performed using MagicPlot software2.9.3. Gaussian peaks were fitted with a maximum error estimation of less than 5%, and correlation coefficients exceeding 0.995 were achieved during the fitting process.

The phase composition of the PTFE coatings was investigated by X-ray diffraction (XRD) using a X’PERT PANalytical diffractometer with CuKα radiation. Bragg–Brentano geometry was employed with a scan step of 0.05° and time per step of 200 s within a 2Θ angle range of 10–30°.

The adhesion of the coatings was assessed through micro scratch testing using a nanoindenter (NanoIndenter XP, Agilent, Santa Clara, CA, USA). A conical tip with a radius of 200 μm was passed progressively across the coated surface under incremental load. A typical normal force variation with sliding distance is shown in Figure S1 (Supporting Information). Both lateral force and penetration depth were recorded during the tests in which a 200 µm scratch length and a maximum load of 300 mN were used. Additionally, for the coating obtained at a pressure of 11 mTorr, a test with a maximum load of 500 mN was conducted. The scratch test was repeated at least three times for each coating. The resulting scratch topography was characterized by the analysing 100 µm × 100 µm atomic force microscopy (AFM) images (Veeco NanoScope Iva, Plainview, NY, USA) obtained in contact mode.

## 3. Results and Discussion

### 3.1. Structure of PTFE Coatings

Three coatings, named here PTFE_3, PTFE_5, and PTFE_11, were obtained by the PED process at 12 kV and nitrogen gas pressures of 3, 5, and 11 mTorr, respectively. Analysing the thickness of the obtained coatings allows for the approximation of the ablation efficiency of the PED process (i.e., whether the conditions used are close to or far from the optimum ablation). This is because optimal deposition in the PED process is defined as the combination of accelerating potential and gas pressure that results in the most efficient ablation of the target, leading to the thickest coatings with the highest deposition efficiency [16]. In the case of the obtained PTFE coatings, as previously observed, the thickness decreased with increasing applied gas pressure without any significant differences in coating roughness: the R_a_ was in the range of 1.5 to 2.5 nm [19]. The thickness of the PTFE_3 coating was approximately 200 nm thick, whereas the PTFE_11 coating was less than 100 nm [19]. The deposition rates for coatings PTFE_3, PTFE_5, and PTFE_11 were 0.42, 0.36, and 0.19 Å/pulse, respectively. Therefore, it can be inferred that the electron beam fluence decreases with increased gas pressure for our processes, and at 11 mTorr, the gas scatters the electrons significantly compared to processes with other pressures.

**Figure 1:**
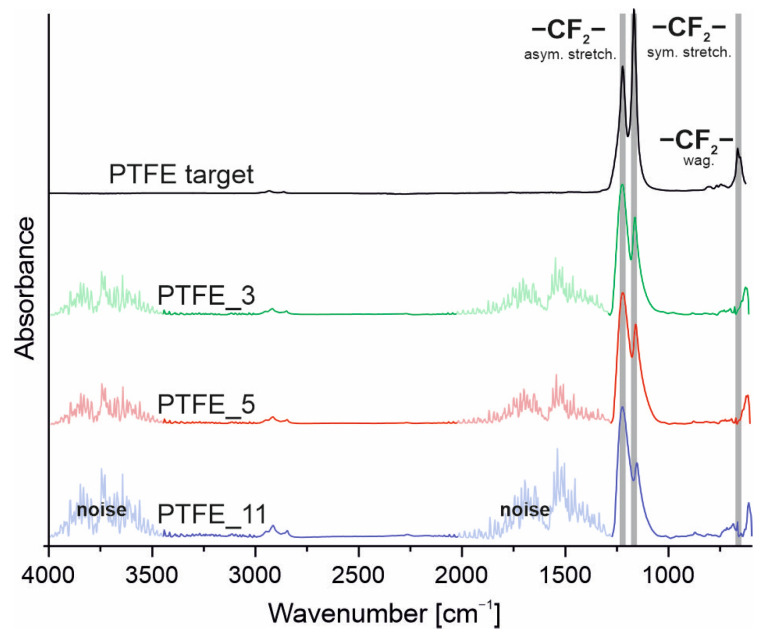
FTIR spectra of the PTFE coatings and target material.

Figure 1 presents the IR spectra of the obtained coatings compared with the spectrum of the starting material (PTFE_target). The observed IR signal noise (originating from the water vapour), which is present in ranges of 4000–3500 cm^−1^ and 2000–1300 cm^−1^, arises primarily from the very low thickness of the coatings, being less than 40% of the volume probed by the IR light [20]. Nevertheless, the characteristic absorption bands of PTFE are distinctively visible, confirming the preservation of the main structure of PTFE.

As seen in Figure 1, the absorption bands of the carbon–fluorine bonds in the wavenumber range 1300–1000 cm^−1^ show slight differences in character. For the coatings, the absorption band broadens towards lower wavenumber values and alters in intensity in proportion to the second band. This observation suggests that the coatings possess different physical structure compared to the starting material.

**Figure 2:**
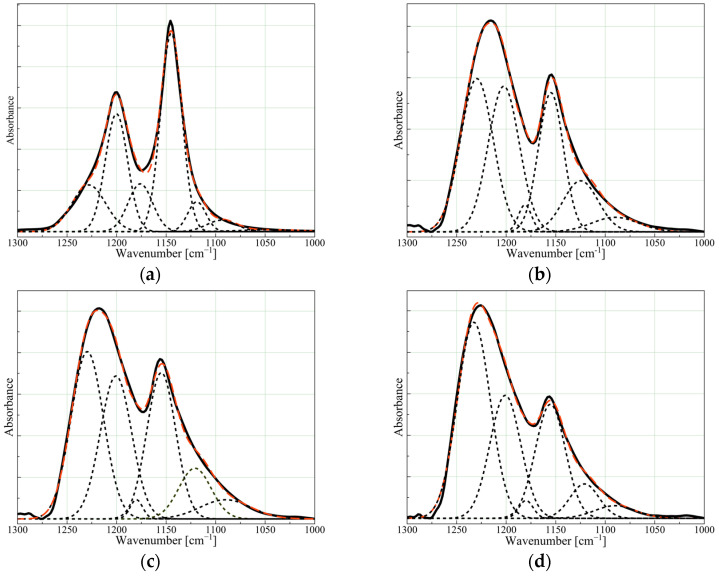
Deconvolution of the IR spectra (black line) in the range 1300–1000 cm^−1^ into individual absorption bands (dashed lines) for (**a**) PTFE_target, (**b**) PTFE_3, (**c**) PTFE_5, and (**d**) PTFE_11. Red dashed line is a fitting curve from individual bands.

A detailed analysis of the 1300–1000 cm^−1^ area of the IR spectrum enables the determination of the proportion of various types of bonds (CF_2_, CF, CC, CF_3_) present in the structure of the coating, and allows the spatial arrangement of the main chain (-(CF_2_)_n_-) along with its relation to the degree of crystallinity of the coating to be assessed. The spatial orientation of the linear parts of the chains can be evaluated by comparing the intensity of the bands with a dipole moment parallel to the axis of the molecule (type A_2_ symmetry) to those perpendicular to the axis of the molecules (type E_1_ symmetry). The ratio of A_2_ to E_1_ bands indicates the dominant spatial arrangement of linear macromolecules (parallel or perpendicular to the coated substrate). The band intensities and proportions can be determined by dividing the analysed area into individual bands using the deconvolution method. Figure 2 and Table 1 present the results of the area deconvolution into individual absorption bands and the percentage of the bands’ area. The table showing the designated areas and maxima of individual bands is presented in the Supporting Information (Table S1).

It is evident from Table 1 that the main bands from CF_2_, at 1201 and 1155 cm^−1^, exhibit a different ratio compared to the PTFE_target, with the predominance of A_2_ symmetry. This indicates a lower number of chains in the coating arranged perpendicular to the substrate. As mentioned previously, in the creation of PTFE coatings using physical methods, the linear chain structure and the resulting high order of the structure are typical. The size discrepancy between C and F atoms makes it impossible to connect subsequent CF_2_ groups in any other way than linearly, with a certain chain torsion forming a helix. Such a chain configuration often forces the chain being created to adopt a perpendicular direction, thus highly ordered PTFE coatings have a predominantly perpendicular chain configuration [9].

For the PTFE coatings, the significantly lower number of perpendicular PTFE chains, as evidenced by the A_2_/E_1_ ratio for the coatings being almost twice as high as for the PTFE_target, indicates a different construction, i.e., they cannot be formed solely as a result of the addition of CF_2_ groups. A significantly higher ratio of C-C bonds (band 1230 cm^−1^) compared to C-F bonds in the structure of the coatings shows that the resulting chains are built with a certain fluorine deficit, leading to chain branching and crosslinking of the coating. The PTFE_3 and PTFE_5 coatings have twice the ratio of C-C bonds compared to the target, while PTFE_11 has almost three times as much. Assuming that the fraction of C-C bonds in the reference PTFE, i.e., 15%, is the initial amount X_0_, while the fraction of C-C bonds in the particular coating X_PTFE_ is the amount enlarged by the number of chain branches and network nodes, the IR-estimated crosslinking degree was calculated based on the formula (X_PTFE_ − X_0_)/X_PTFE_. On this basis, it can be inferred that the degree of crosslinking of the PTFE_3 and PTFE_5 coatings is at a similar level, while PTFE_11 exhibits the highest degree of crosslinking. Since an increase in the degree of crosslinking of the polymer material directly affects its degree of crystallinity (lowering it due to the disruption of the symmetry and linearity of the chains required for the formation of ordered crystalline regions [23]), an examination of the physical structure was carried out using the XRD method to confirm the interpretation of the chemical structure of the coatings.

**Figure 3:**
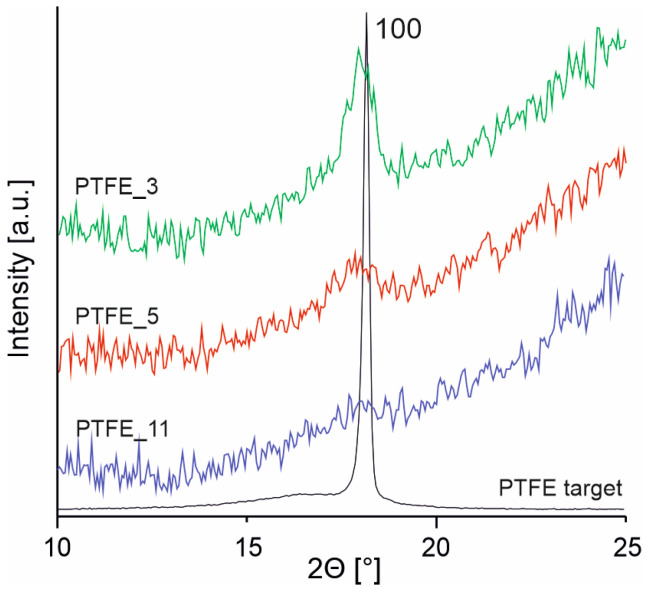
X-ray diffraction patterns for target material and PTFE coatings.

Figure 3 shows diffraction patterns in the angular region 2θ = 10–25° of the PTFE target and the coatings. In general, PTFE crystals at room temperature have a hexagonal symmetry consisting of chains built by CF_2_ groups forming a helix. A typical diffraction peak of the hexagonal phase corresponding to plane 100 occurs at 2θ = 18° [24] and is clearly seen in the diffraction pattern of the PTFE target. For the PTFE coatings, the Bragg reflection 100 intensity is very low and decreases with increasing gas pressure used in the PED process, suggesting amorphousness of the coatings. The XRD results are in line with the interpretation of IR spectra and the description of the chemical structure of the coatings, also indicating that materials are disordered probably due to chemical crosslinking, but it should be considered that the decreasing thickness of the coatings may also have an influence on the XRD pattern. Nevertheless, the increased amorphousness of the coatings will have a direct impact on its mechanical properties.

### 3.2. Mechanical Behaviour of PTFE Coatings

**Figure 4:**
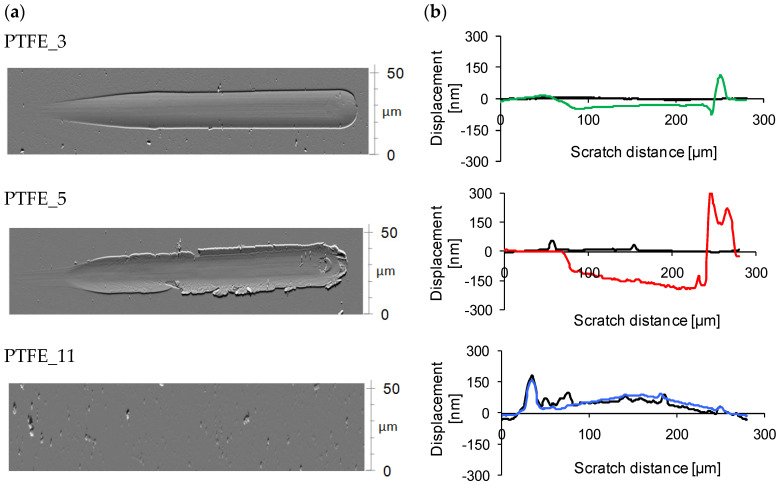
(**a**) AFM topography of scratch area; (**b**) the depth profiles along the scratch length for coatings. Black line—surface profile before scratch; coloured line—surface profile after scratch. Maximum test load for PTFE_3 and PTFE_5 was 300 mN, and for PTFE_11, 500 mN.

Figure 4 shows AFM images and the graph of coating surface profiles after scratch testing. The behaviour of the coatings during the tests varied significantly depending on the pressure of the gas used during the deposition. In general, for small loads (up to approximately 60 mN) during the test, all coatings demonstrated elastic deformation. This is indicated by the surface profile after the scratch test following the same profile as before the scratch test. However, for coatings obtained with lower gas pressure (PTFE_3 and PTFE_5), plastic deformation occurred at loads above approximately 60 mN (at about 80 µm from the beginning of the test), as indicated by a negative deviation from the original profile before scratching. PTFE_11 showed a complete elastic recovery of the deformed material at all scratch loads.

**Figure 5:**
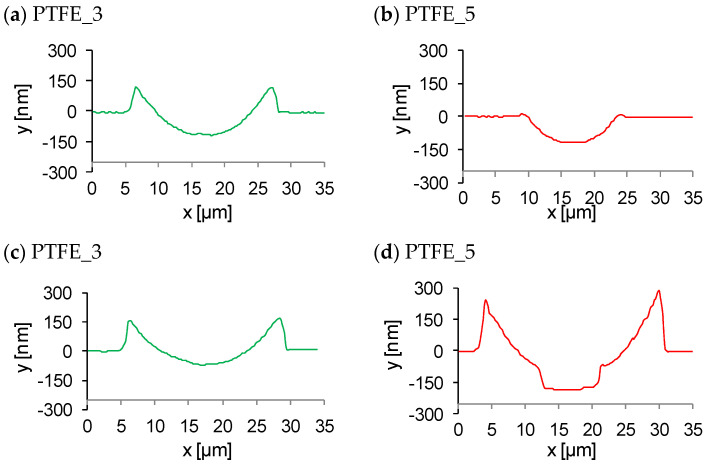
Depth profiling of scratch cross-sections for the coating PTFE_3 (**a**,**c**) and PTFE_5 (**b**,**d**). Profiles obtained at a distance of ca 80 μm (**a**,**b**) and ca 235 μm (**c**,**d**) from the beginning of the scratch test; profiles were made using a nanoindenter.

In the coating PTFE_3, plastic deformation is observed without any visible cracks. The coating material has been pushed aside during tip movement. It can be observed that the pile-up size is comparable with the groove size (Figure 5a). A part of the coating material was also piled up at the end of the scratch groove. The maximum depth of the groove is much smaller than the total thickness of the coating, which indicates that elastic deformation contributes to the total deformation. No signs of coating delamination were registered. It can be concluded that, for this coating, the cohesive strength is significantly smaller than the coating–substrate adhesion strength and that stress relaxation under loading occurs only by coating deformation. A susceptibility to shear-driven deformation (due to low cohesion) is a typical behaviour of PTFE material [24], but the partial elastic recovery is probably a result of crosslinking of the coating.

In the case of the PTFE_5 coating, the formation of a groove is also observed for a load greater than 60 mN. However, the mechanical response of the coating material is different from that for PTFE_3. Groove formation at the beginning is not accompanied by such a substantial pile-up, as observed for PTFE_3 (Figure 5b). Further increase in the load during the scratch leads to pile-up of material and the size of the pile-up increases with the length of the scratch. The biggest pile-up is formed at the end of the scratch (Figure 5d) and its volume is significantly larger than that recorded for PTFE_3 (Figure 5c). The volume of the material accumulated at the end of the scratch track is also significantly greater in this case (Figure 4b). Moreover, the material which was pushed aside is unevenly distributed and numerous breaks in its continuity can be observed (Figure 4a). At the end of the scratch, a delamination of the coating from the substrate is present.

The depth of the groove increases with scratch length and reaches the end of the depth corresponding to the coating thickness (Figure 5d). In the case of the PTFE_5 coating, it appears that, during the scratch test, the coating material stacks at the stylus tip front and is gradually deposited on both sides of the scratch track and at its end. It can be assumed that the cohesive strength of the coating is similar to the forces of adhesion.

The greatest difference in the mechanical behaviour of the coating was observed during the scratch test where the coating was obtained at the highest pressure, PTFE_11. After a scratch test of up to 300 mN load, no change on the surface of the coating was observed. Therefore, for these tests, a further scratch test with maximum load of 500 mN (maximum normal load available on the device) was applied. However, this also did not result in any significant changes on the surface. The surface profile registered before the test corresponded to the profile obtained after the test (Figure 4). There were also no changes to the surface observed in the AFM analysis (Figure 4). It can be assumed that the deformation occurring during the scratch test was fully elastic, causing the surface to return to its initial state. This behaviour of the coating material, a rubber-like one, would indicate that the applied strain range is located in an elastic region of deformation, which further suggests a significantly higher yield strength of this material. These features can be related to the crosslinked structure of the PTFE_11.

### 3.3. Structural Modifications of PTFE under High-Energy Radiation and PED Process Conditions

One might initially find it unexpected that the coating obtained under the highest gas pressure (11 mTorr), resulting in the lowest fluence, exhibits the highest degree of crosslinking, as evidenced by the increased ratio of C-C bonds and enhanced amorphousness of the material. However, a potential explanation for the structure of the PTFE_11 coating can be based on the structural changes observed in the PTFE material under the influence of energy input, whether this is from heat, electromagnetic radiation, or a high-energy beam such as protons.

**Figure 6:**
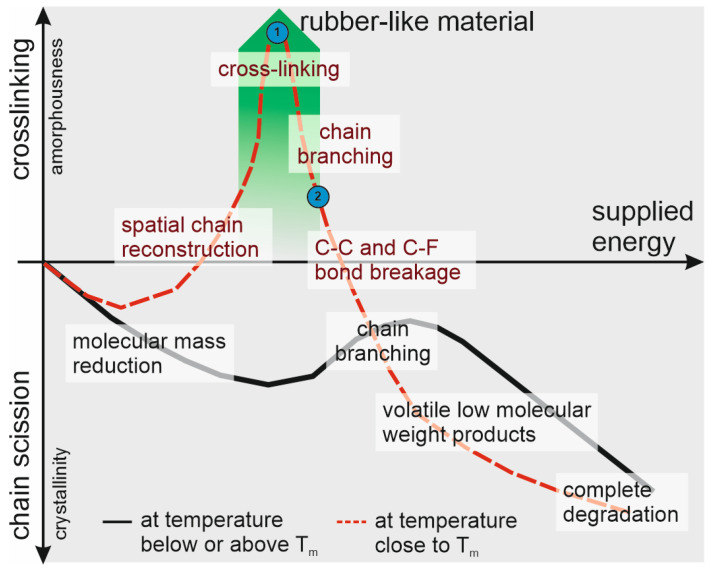
Type of structural change of PTFE as a function of the supplied energy. Blue circles represent hypothetical conditions corresponding to those during the coating obtaining process: 1—PTFE_11 and 2—PTFE_5 and PTFE_3.

In general, when polymers are exposed to a sufficiently high level of energy, they undergo either chain scission or crosslinking, or a combination of both, with one phenomenon typically prevailing. The dominant process is largely determined by the type of bonds present in the polymer’s structure. Regardless of the type of electromagnetic radiation or high-energy beam, the structural changes in PTFE follow a similar pattern. While the intensity of these changes and the required energy levels may vary, the underlying nature remains consistent. Figure 6 provides our interpretation in a graphical representation of the changes in PTFE material, illustrating the type of structural change as a function of the supplied energy, independent of the specific external energy source.

For PTFE, due to the difference in C-F and C-C bond energies and the specific helical chain conformations, chain scission is the primary mechanism within this material [25]. Initially, at lower energy doses, the formation of free radicals and subsequent chain scission predominantly takes place in amorphous regions of semicrystalline PTFE material. The shortening of PTFE chains enhances their mobility and ability to organize, resulting in a lower molecular weight, but an increased crystallinity and hardness of the material [26,27]. With increasing energy dose, a higher quantity of free radicals is formed, increasing the probability of their interaction and leading to PTFE chain branching [28]. Branching competes with the chain scission (thus ordering of shorter highly mobile chains), affecting the degree of crystallinity and the mechanical properties of the material. The extent of chain branching is influenced by the type of beam and the irradiation temperature [29,30]. Further elevation of the radiation energy dose results in extensive bond breakage, including stochastic breaking of C-C and C-F bonds, ultimately leading to the formation of low-molecular-weight volatile products, fluorine radicals, and complete material degradation [26].

As already mentioned, the extent of the chain branching process is influenced by the temperature of the PTFE material under radiation. An exceptional situation occurs when the temperature approaches the melting point of PTFE. Around the melting point, as a result of the breaking of physical interactions, an increased mobility of PTFE macromolecules takes place. If this is combined with a sufficiently large dose of energy to generate free radicals, without surpassing the threshold for intensive material degradation, the branching and further crosslinking process starts to dominate changes in the PTFE structure (Figure 6, red dashed line, point no. 1). Under these conditions, the PTFE structure exhibits spatial chemical crosslinking, heightened amorphousness, and the mechanical properties show a similarity to those of elastomeric materials. The behaviour of the PTFE material becomes fully rubber-like. However, achieving such a structure requires precise control over the temperature range and energy level of the applied beam, which are constrained within a narrow range [30,31,32,33].

Given that the structure of the coatings obtained, particularly that of PTFE_11, bears resemblance to structures formed during radiation at the material’s melting temperature—namely, crosslinked with rubber-like properties—we hypothesize that the conditions applied in the PED process corresponded to the region of branching/crosslinking dominance indicated in Figure 6 by the green area. Furthermore, we propose that alterations in the PED process parameters largely mirror the specificity of transformations described by the conditions along the red dashed line.

**Figure 7:**
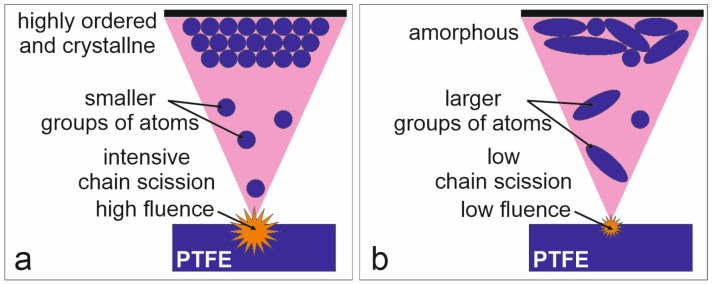
Schematic visualization of differences in the coatings creation at higher (**a**) and lower (**b**) electron beam fluences.

Lower gas pressure (closer to the optimal PED process conditions) results in a higher fluence of the electron beam, likely corresponding to the region where bond breaking is more intense (region marked by blue circle no. 2). High electron beam fluence leads to enhanced bond breakage at the ablation step. Consequently, the formation of shorter PTFE chains (smaller groups of atoms) transported in the plasma plume to the substrate results in fewer crosslinked structures (PTFE_3 and PTFE_5). Creation of the coating from groups consisting of one to a few carbon atoms allows a highly ordered and crystalline PTFE structure to form, as typically obtained for other PVD techniques [1,34,35], as schematically shown in Figure 7a. The high mobility and low steric hindrance of the deposited short PTFE chain fragments promote the creation of a linear PTFE chain.

Conversely, reducing fluence (achieved by increasing gas pressure) decreases the intensity of chain scission (region marked by blue circle no. 1), likely resulting in the ablation of larger groups of atoms (longer chains). These longer chain elements deposited onto the substrate exhibit increased steric hindrance, precluding the formation of a highly ordered structure and promoting branching and network creation to occur (schematically shown in Figure 7b). As a result, the coating then adopts an amorphous structure and exhibits rubber-like behaviour (PTFE_11).

## 4. Conclusions

In this study, three PTFE coatings with different mechanical responses under scratch test conditions were obtained using pulsed electron beam deposition at 12 kV and various nitrogen pressures. Particularly noteworthy is the PTFE coating deposited at the highest gas pressure of 11 mTorr, which exhibited rubber-like properties. The analysis results and our hypothesis suggest that, under conditions of high gas pressure in the PED method, limited chain scission occurs during ablation due to low fluence. Consequently, large chain fragments are transported to the substrate with restricted ability to rebuild the ordered (crystalline) structure of PTFE (as typically observed under conditions of high fluence where intensive chain scission occurs). In this way, low fluence leads to the formation of a coating with an increased amorphous structure, where the presence of numerous radicals resulting from electron beam interaction during ablation promotes crosslinking. The findings of this research indicate that by selecting appropriate deposition conditions, including the working gas pressure, the physical structure of the coating material in the PED method can be significantly modified, thereby influencing its properties such as mechanical behaviour.

## Figures and Tables

**Table 1:** The percentage of the individual bands’ area calculated from the IR region deconvolution.

Wavenumber [cm^−1^] (Bond)	PTFE Target	PTFE3	PTFE5	PTFE11
1230 (C-C)	15.0	31.6	31.9	41.8
1201 (CF_2_, A_2_)	25.1	28.6	26.4	24.6
1180	11.2	2.2	1.4	1.6
1155 (CF_2_, E_1_)	40.2	22.0	24.9	22.1
1121	5.2	11.3	9.7	6.4
1090 (C-F, branching)	3.3	4.3	5.7	3.4
1209/1153 (A2/E1)	0.6	1.3	1.1	1.1
IR-estimated crosslinking degree *	-	0.52	0.53	0.64

* The obtained values represent the increase in the fraction of C-C bonds in the structure, which correlates with the degree of crosslinking of the PTFE material, and are calculated for comparison purposes rather than indicating the actual degree of crosslinking of the coating.

## Data Availability

Data presented in this study are available upon request from the corresponding author due to the fact that the data are a part of ongoing research and future project research. As such, controlled access to the data ensures that they are not prematurely disclosed or misinterpreted before additional analyses or publications are completed. We are committed to transparency and scientific rigor and welcome inquiries regarding the data. Please contact the corresponding author for further information or access to the data.

## Supplementary Materials

The following supporting information can be downloaded at: https://www.mdpi.com/article/10.3390/polym16091205/s1, Figure S1: Normal force variation during scratch tests versus sliding distance. Increase in load began after 40 µm and the maximum value of 300 mN was reached 40 µm before the end of the tip movement; Table S1: Designated areas and maxima of individual bands achieved by the IR spectra deconvolution.
