# Supplementary material for: Rubber-like PTFE Thin Coatings Deposited by Pulsed Electron Beam Deposition (PED) Method
Source: Polymers (Basel). 2024 Apr 25;16(9):1205. doi: 10.3390/polym16091205 (PMC11085439; doi:10.3390/polym16091205)
Supplement: Supplementary file 1 [file polymers-16-01205-s001.zip › polymers-2960768-supplementary.pdf]

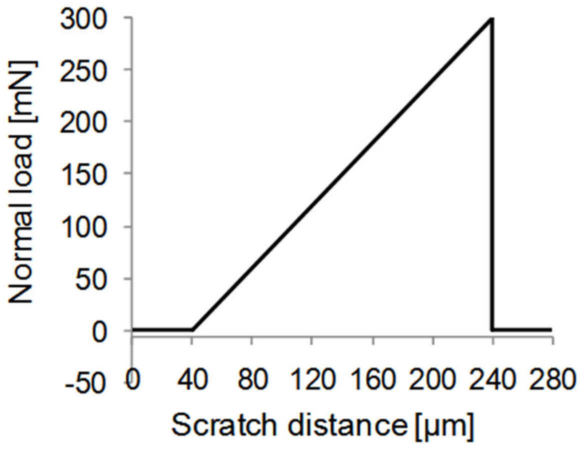

Figure S1. Normal force variation during scratch tests versus sliding distance. Increase of load began after 40 µm and the maximum value of 300mN was reached 40 µm before the end of the tip movement.

Table S1. Designated areas and maxima of individual bands achieved by the IR spectra deconvolution.

PTFE_11			PTFE_5			PTFE_3			PTFE_target		
**Band max. [cm^{-1}]**	area	Band ratio [%]	Band max. [cm^{-1}]	area	Band ratio [%]	Band max. [cm^{-1}]	area	Band ratio [%]	Band max. [cm^{-1}]	area	Band ratio [%]
**1233**	3.5401	41.8	**1230**	5.8547	31.9	**1230**	6.3013	31.6	**1228**	9.6614	15.0
**1201**	2.0807	24.6	**1201**	4.8498	26.4	**1202**	5.6943	28.6	**1200**	16.1484	25.1
**1180**	0.1392	1.6	**1180**	0.2611	1.4	**1181**	0.4458	2.2	**1176**	7.1776	11.2
**1155**	1.8740	22.1	**1155**	4.5680	24.9	**1155**	4.3807	22.0	**1145**	25.8377	40.2
**1121**	0.5443	6.4	**1121**	1.7719	9.7	**1125**	2.2486	11.3	**1119**	3.3476	5.2
**1090**	0.2853	3.4	**1090**	1.0533	5.7	**1090**	0.8488	4.3	**1094**	2.1037	3.3
